# Role of Aspartate in Immune Response and Mortality in a Polymicrobial Sepsis Model: Insights from Metabolomics and Transcriptomics

**DOI:** 10.3390/cells15060513

**Published:** 2026-03-13

**Authors:** Min Ji Lee, Bo Mi Kim, Se Rin Choi, Seongmin Kim, Ye Jin Park, Yun-Seok Kim, Kihwan Choi, Chang June Yune, Tae Nyoung Chung, Jinkun Bae, Nam Joo Yun, Jiwon Jeon, Han A Reum Lee, Jiewan Kim, Dong-Hyuk Kim, Ji Heon Noh, Chungoo Park, Sangchun Choi, Choong Hwan Lee, Kyuseok Kim

**Affiliations:** 1 Department of Emergency Medicine, CHA University School of Medicine, Seongnam 13496, Republic of Korea; minji.lee29@gmail.com (M.J.L.); alfks4050@naver.com (B.M.K.); yejin6577@naver.com (Y.J.P.); kh4213@gmail.com (K.C.); hendrix74@cha.ac.kr (T.N.C.); galen97@chamc.co.kr (J.B.); yosi0708@naver.com (N.J.Y.); tw7682@nate.com (J.J.); harlee91@naver.com (H.A.R.L.); jiewankim@gmail.com (J.K.); 2 Department of Bioscience and Biotechnology, Konkuk University, Seoul 05029, Republic of Korea; csr0701@gmail.com (S.R.C.); walking99@hanmail.net (D.-H.K.); 3 School of Biological Science and Technology, Chonnam National University, Gwangju 61186, Republic of Koreachungoo@jnu.ac.kr (C.P.); 4 Department of Biochemistry, Chungnam National University, Daejeon 34134, Republic of Korea; journi@cnu.ac.kr; 5 Emergency Department, Soonchunhyang University College of Medicine, Asan 31538, Republic of Korea; avenue59@schmc.ac.kr

**Keywords:** sepsis, immune modulation, omics study, immune suppression, aspartate

## Abstract

Sepsis is a life-threatening syndrome characterized by dysregulated host responses to infection. In addition to early hyperinflammation, many patients develop profound immune suppression, and multiple targeted immunotherapies have failed to improve outcomes, highlighting the need for actionable biomarkers and new therapeutic strategies. Here, we integrated metabolomic and transcriptomic profiling of peripheral blood mononuclear cells (PBMCs) and splenocytes in rat models of polymicrobial sepsis to identify metabolites associated with immune dysfunction. Candidate findings were validated using in vivo supplementation studies and in vitro functional assays, and clinical relevance was assessed in PBMCs from patients with sepsis and healthy volunteers. Across omics datasets, intracellular aspartate (ASP) was consistently reduced in immune cells during sepsis and was associated with features of immune paralysis. Supplementation with L-ornithine L-aspartate (LOLA), an ASP source, improved survival in septic rats, enhanced bacterial clearance, and mitigated acute kidney injury. In vitro, pharmacologic or genetic disruption of ASP production impaired phagocytosis and cytokine responses, which were partially rescued by ASP supplementation. Consistently, patients with sepsis exhibited lower intracellular ASP levels in PBMCs than healthy volunteers. Together, these results support a critical role for ASP in maintaining immune competence during sepsis and suggest that intracellular ASP may serve as a biomarker of immune suppression and a potential therapeutic target.

## 1. Introduction

Sepsis is defined as a life-threatening organ dysfunction caused by dysregulated host responses to infection [1]. Worldwide, it has a high incidence and mortality and continues to be a public health problem [2,3,4]. Given this background, the World Health Organization (WHO) has announced sepsis as a global health priority [5]. Despite extensive drug-development efforts, no targeted therapy has consistently improved clinical outcomes beyond supportive care.

The immunopathology of sepsis is heterogeneous and dynamic. Alongside hyperinflammation, many patients develop early and sustained immune suppression (immune paralysis), which is associated with secondary infections and mortality [6,7]. Immunoadjuvant strategies such as PD-1/PD-L1 blockade and interleukin-7 have shown promise in preclinical studies, but clinical translation has been challenging, partly because immune status is rarely measured in real time and suitable biomarkers are limited [8,9].

Previously, we investigated the serial change of immune suppression in polymicrobial sepsis model and found that immune suppression in PBMCs and splenocytes start earlier in sepsis, and it is most profound in 1–2 days after sepsis, which recover 3 to 5 days after sepsis [10].

Amino acids support immune-cell activation by providing bioenergetic substrates, biosynthetic precursors, and redox balance. Aspartate participates in nucleotide and protein synthesis and connects mitochondrial metabolism to cytosolic biosynthetic pathways [11,12]. Although ASP metabolism has been studied in cancer biology, its role in sepsis-associated immune dysfunction has not been well characterized.

We hypothesized that intracellular Asp deficiency contributes to immune suppression in a polymicrobial sepsis model and that restoring Asp availability could improve immune function and outcomes. To test this hypothesis, we performed metabolomic and transcriptomic profiling in rat sepsis models, conducted mechanistic validation experiments in vivo and in vitro, and evaluated intracellular Asp levels in PBMCs from patients with sepsis.

## 2. Materials and Methods

### 2.1. Animals

Male Sprague-Dawley rats (270–330 g) were used to reduce phenotypic variability. Animals were housed at 20–24 °C with free access to standard chow and water for at least 7 days before experiments. Con

### 2.2. In Vivo Sepsis Model

All animal procedures were approved by the Institutional Animal Care and Use Committee of CHA University (IACUC 230143), adhered to National Institutes of Health guidelines, and were reported in accordance with the ARRIVE guidelines. Polymicrobial sepsis was induced using a weight-adjusted cecal slurry peritonitis model, as previously described [13]. Briefly, donor rats were anesthetized with intramuscular tiletamine/zolazepam (Zoletil, 50 mg/kg) and xylazine (10 mg/kg). After midline laparotomy, fecal material was collected from the cecum, weighed, and diluted in 5% dextrose saline (1:3). Recipient rats underwent laparotomy under identical anesthesia and received an intraperitoneal injection of vortexed slurry at a volume adjusted to body weight. All animals received subcutaneous fluid resuscitation (30 mL/kg of 5% dextrose saline) and imipenem (25 mg/kg, subcutaneously, twice daily for 2 days). Sepsis was confirmed by decreased activity, lethargy, shivering, and piloerection [13].

Experiment 1: Endotoxin tolerance (ex vivo study). Endotoxin tolerance through ex vivo PBMCs/splenocytes 24 h after sepsis induction was done with stimulation with LPS.

Experiment 2: Omics study. In the omics study, we introduced cecal slurry model with 5.5 mL/kg cecal slurry. The rats were randomly assigned to the study group at 6 h, 24 h, and 5 days. The stratified randomization by weight was performed by an assistant in the experiments. We sacrificed the animals at each allocated time points, and PBMCs were isolated. If the animals could not survive until the allocated time points, they were excluded from the analysis. During the observation period, an employee of the animal research center monitored the animals twice per day. If the animal was doomed to die, the research team would take notice and decide to execute euthanasia.

Experiment 3: Survival study. In the survival study, we introduced two serial doses of cecal slurry model with 5.0 mL/kg cecal slurry at 48 h intervals. L-Ornithine L-Aspartate (LOLA) was injected twice per day for 3 days. It was injected via the tail vein at 24 h/48 h time points, and intraperitoneal injection at other time points. Survival was monitored every 12 h for 14 days. During the observation period, an employee of the animal research center monitored animals twice per day, and if the animal seemed close to death, they notified the research team, who decided to carry out euthanasia.

Experiment 4: CFU, Blood chemistry, and tissue study. In this study, we administered LOLA twice per day for 2 days. Blood and tissues were harvested 24 h after the second fecal slurry administration. The colony-forming unit (CFU) in the blood and spleen was counted. We measured serum creatinine, ALT, plasma lactate, glucose, arterial blood gases, and electrolytes.

Investigators performing outcome measurements were blinded to the treatment group whenever feasible. Animals that died before their pre-specified sampling time point were excluded from time-point analyses but were included in survival analyses.

### 2.3. Metabolomics Study

#### 2.3.1. Extraction of Sample for Metabolomics

The PBMCs and splenocytes samples were extracted by adding 1 mL of ice-cold 100% methanol and 10 L internal standard solution (2-chlorophenylalanine, 1 mg/mL in water) as described by [14]. Frozen spleen tissue (100 mg) was extracted similarly [15]. Samples were homogenized (30 Hz, 10 min; Retsch GmbH & Co, Haan, Germany), centrifuged (12,000 rpm, 10 min, 4 °C), and supernatants were filtered through 0.2 µm PTFE syringe filters. Extracts were dried under vacuum and stored at −80 °C. Prior to analysis, dried extracts were reconstituted in methanol (10 mg/mL) and re-dried for two-step derivatization.

#### 2.3.2. Gas Chromatography–Time-of-Flight–Mass Spectrometry Analysis

Gas chromatography–time-of-fight–mass spectrometry (GC-TOF-MS) analysis was performed on an Agilent 7890A gas chromatograph coupled to an Agilent 7693 autosampler (Agilent, Atlanta, GA, USA) as previously described [16]. Re-dried extracts were oximated with 50 µL methoxyamine hydrochloride in pyridine (20 mg/mL; 90 min, 30 °C) and silylated with 50 µL MSTFA (30 min, 37 °C). Quality-control (QC) samples were prepared by pooling equal aliquots (10 µL) from each extract. One microliter of derivatized sample was injected in splitless mode. Injector and ion-source temperatures were 250 °C and 230 °C, respectively. The oven was set at 75 °C for 2 min, ramped to 300 °C at 15 °C/min, and these conditions were held for 3 min. Data were acquired at 10 scans/s over 50–1000 m/z. Analytical runs were performed in blocks of eight samples with intermittent QC injections.

#### 2.3.3. UHPLC–LTQ–Orbitrap–MS/MS Analysis

The analysis was performed using a UHPLC system equipped with a Vanquish binary pump H system (Thermo Fisher Scientific, Waltham, MA, USA) coupled with an auto-sampler and column compartment. Chromatographic separation was performed on a Phenomenex KINETEX^®^ C18 Column (100 mm × 2.1 mm, 1.7 m; Torrance, CA, USA), and the operational parameters were adapted from a study by Lee et al. [17]. Samples were analyzed in randomized blocks of eight with intermittent pooled-QC injections to minimize systematic drift.

#### 2.3.4. LC–Triple-Quadrupole-MS Analysis

Targeted Asp analysis was conducted using a Nexera2 LC system (Shimadzu Corp., Kyoto, Japan) coupled to a triple-quadrupole mass spectrometer (LC-MS 8040, Shimadzu, Corp., Kyoto, Japan) with an electrospray source. Five microliters were injected into a HSS T3 column (100 × 2.1 mm, 1.8 µm, Waters, Milford, MA, USA). Mobile phases were 0.1% formic acid in water (A) and 0.1% formic acid in acetonitrile (B) at 300 µL/min. The gradient was 5% B for 1 min and increased linearly to 100% over 7 min, with a total run time of 9 min. MRM transition for Asp was m/z 134.0 → 74.0.

#### 2.3.5. Data and Statistical Analysis

MS data processing and multivariate statistical analysis were conducted as previously described [16]. Raw GC-TOF-MS data were converted to NetCDF using ChromaTOF (version 4.44, LECO), and processed using Metalign to generate a matrix of retention times, accurate masses, and normalized intensities. Multivariate analyses (PCA and PLS-DA) were performed in SIMCA-P+ (version 12.0, Umetrics, Umea, Sweden). Discriminant metabolites were selected using variable importance in projection (VIP) > 0.7 and *p* < 0.05. Metabolites were annotated by retention time and mass-fragment matching against in-house libraries and public databases, including the NIST database (Version 2.0, 2011 FairCom; Gaithersburg, MD, USA), and the Human Metabolome Database (HMDB).

### 2.4. Transcriptomics Study

The collected splenocytes were isolated and total RNA from each sample was extracted using Trizol reagent (Invitrogen, Carlsbad, CA, USA), and quality control and quantification were performed by a Bioanalyzer 2100 system (Agilent Technologies, Santa Clara, CA, USA) and Nanodrop ND-2000 Spectrophotometer (Thermo Scientific, Waltham, MA, USA). For RNA-sequencing (RNA-seq), the construction of libraries from total RNAs was performed using the NEBNext Ultra II Directional RNA-Seq Kit (NEW ENGLAND BioLabs, Inc., Hitchin, UK) according to the manufacturer’s instructions. Briefly, the isolated mRNAs were used to ligate the adaptors, and then cDNA was synthesized using reverse-transcriptase with adaptor-specific primers. PCR was performed for library amplification, and subsequently, libraries were checked for quality, quantification, and size distribution using the TapeStation HS D1000 Screen Tape (Agilent Technologies, Santa Clara, CA, USA) and using a StepOne Real-Time PCR System (Life Technologies, Inc., Carlsbad, CA, USA). High-throughput sequencing was performed as paired-end 101 base pair reads on a NovaSeq 6000 (Illumina, Inc., San Diego, CA, USA).

All raw sequence reads were preprocessed to remove bases with low quality and adapter sequences using Trimmomatic (version 0.39) (Insiligogen, Inc., Yongin-si, Republic of Korea) [18]. The rat reference genome (in FASTA format), HISAT2 genome index file (Rnor_6.0), and annotation (in GTF format) containing transcript structures were obtained from the Ensembl database [19]. The cleaned paired-end reads were aligned to the reference genome using HISAT2 (v2.2.1) [20] with default parameters. The generated BAM (binary alignment map) files from the previous step were further processed with StringTie (v2.2.1) and prepDE [21] to quantify transcript abundances using the transcripts per kilobase million (TPM) and counts per million mapped reads (CPM) normalizations. Differential expression analysis was carried out using edgeR package (v3.38.4) [22]. In this study, we defined genes as significantly differentially expressed if they had read counts greater than 10 in at least one sample and a false discovery rate of lower than 1% (FDR; q-value). We performed the enrichment analysis of Gene Ontology (GO) categories and Kyoto Encyclopedia of Genes and Genomes (KEGG) pathway analysis of DEGs using DAVID (the Database for Annotation Visualization and Integrated Discovery; v2023q4 released; https://davidbioinformatics.nih.gov/, accessed on 9 March 2026) functional annotation tool [23].

### 2.5. CFU Assay

Blood and spleen samples were used to quantify bacterial load. Spleen tissue (30 mg) was homogenized in 500 µL DPBS, centrifuged (12,000 rpm, 10 min, 4 °C), resuspended in 1 mL DPBS, and diluted 1:10. For blood, 200 µL was diluted into 800 µL DPBS. Dilutions were plated on TSA agar without antibiotics and incubated overnight at 37 °C before colony counting.

### 2.6. Intracellular ASP Measurement

Intracellular Asp levels were quantified using a colorimetric assay kit (Ab 102512, Abcam, Waltham, MA, USA). Cell lysates were obtained by centrifuging at 14,000× *g* for 15 min at 4 °C, and supernatants were incubated with the reaction mix protected from light. Absorbance was read at 570 nm, and Asp concentration was calculated from a standard curve.

### 2.7. Phagocytosis Study

The phagocytic activity of PBMCs was assessed using *E. coli* particles (E2861, Invitrogen, Carlsbad, CA, USA) and an opsonization kit (E2870, Invitrogen, Carlsbad, CA, USA) according to the manufacturer’s instructions. PBMCs were incubated with *E. coli* particles for 1.5 h at 37 °C. Subsequently, cells were washed twice with cold DPBS and fixed with 4% paraformaldehyde for 10 min at room temperature. After removing the supernatant, cells were washed twice and resuspended in FACS buffer (0.5% BSA, 2 mM EDTA). Following immediate incubation, cells were analyzed using flow cytometry with CytExpert software (version 2.4.0.84, Beckman, Brea, CA, USA).

### 2.8. Biochemistry

Serum alanine aminotransferase (ALT), blood urea nitrogen (BUN), and creatinine were measured using an automated chemistry analyzer (Cobas c502, Roche, Basel, Switzerland). Plasma glucose, albumin, and electrolytes were measured from arterial blood (ABL90 Flex Plus, Radiometer, Copenhagen, Denmark).

### 2.9. Ex Vivo Endotoxin Tolerance Assay

Endotoxin tolerance was measured through ex vivo PBMCs, and splenocytes were isolated after induction of sepsis. PBMCs were isolated using the Ficoll-Paque PLUS gradient method [24]. Isolated PBMCs were stimulated with LPS to observe and compare the levels of immune paralysis. PBMCs were seeded at a density of 1 × 10^5^ cells/well in 96-well plates, and 100 ng/mL of LPS (Escherichia coli O26:B6, L2762, Sigma-Aldrich, St. Louis, MO, USA) was added to each well. After 5 h, the culture medium was collected. Splenocytes were isolated and stimulated with LPS to compare immune paralysis [25]. Isolated splenocytes were seeded at a density of 5 × 10^5^ cells/well in 6-well plates, and 1 μg/mL LPS was added to each well. After 5 h, the culture medium was collected.

### 2.10. THP-1 Endotoxin Tolerance Model

To estimate the immune-enhancing effect of both LOLA and ASP, THP-1 cells (ATCC, Manassas, VA, USA) were seeded at a density of 1 × 10^5^ cells per well in 48-well plates with LPS and either LOLA or ASP. Cells were incubated at 37 °C with 5% CO_2_. For the endotoxin tolerance model, cells were stimulated with LPS at a concentration of 10 ng/mL for 4 h. Following this, LPS was removed, and the cells were allowed to rest for 16 h in fresh RPMI medium (L0498, Biowest, Nuaillé, France). Subsequently, re-stimulation was carried out with the same concentration of LPS for 4 h.

### 2.11. qPCR Study

Total RNA was extracted from rat splenocytes using TRIzol reagent (15596018, Invitrogen, Waltham, MA, USA). Then, RNA was reverse-transcribed into complementary DNA using RT PreMix, RNase H Minus (dT20) (K-2241, BIONEER, Daejeon, Republic of Korea) according to the manufacturer’s instructions. qPCR was performed using qPCR master mix (K-6253, BIONEER, Daejeon, Republic of Korea) with CFX connect Real-Time System (BIORAD, Hercules, CA, USA). The relative mRNA levels were calculated by the 2^−ΔΔCt^ method with normalization to the reference gene β-actin. The primer used in this study was as follows: Got1; forward: 5′-ACCACGAGTACTTGCCCATC-3′, reverse: 5′-CATCGCCCTAAGAAGTCAGC-3′, Got2; forward: 5′-CCAAGACTTGCGGCTTTGAC-3′, reverse: 5′-CTTTTTCTTCACCACCGCCG-3′, β-actin; forward: 5′-TGTGGATTGGTGGCTCTATC-3′, reverse: 5′-AGAAAGGGTGTAAAACGCAG-3′.

### 2.12. Western Blot Analysis

The splenocytes were isolated by cell strainer (#352350, Falcon corning, Corning, NY, USA) from rat spleen and lysed by RIPA buffer with a protease inhibitor and phosphatase inhibitor (#1862209 and 1862495, Thermo Fisher Scientific, Waltham, MA, USA). Each loading sample had 20 μg protein and was separated by 12% SDS-PAGE. Then, the gels were transferred to PVDF membranes (#10600023, cytiva, Amersham, England) and blocked in 5% BSA for 1 h at room temperature. The membranes were incubated with a primary antibody (GOT1 Rabbit pAb [1:1000, #A5822, ABclonal Technology, Boston, MA, USA], GOT2 Polyclonal Antibody [1:1000, #PA5-27572, Invitrogen, Waltham, MA, USA], β-actin (C4) mouse monoclonal IgG1 [1:1000, #sc-47778, Santa Cruz Biotechnology, Dallas, TX, USA]) overnight at 4 °C and with secondary antibody (Goat Anti-Rabbit IgG antibody (HRP) [1:10,000, #GTX213110-01, GeneTex, Irvine, CA, USA], Mouse IgG antibody (HRP) [1:10,000, #GTX213111-01, GeneTex, Irvine, CA, USA]) for 1 h at room temperature. After attaching the antibody, the membranes were washed 6 times for 30 min with TBST. The protein expressions were visualized using ECL solution (GTB5060, GeneSTAR, Songpa, Seoul, Republic of Korea) with a gel documentation system (G:BOX ChemiXX6, Syngene, Mumbai, India) and the band intensities were quantified by Image J progra. (version 1.54).

### 2.13. In Vitro M1 Macrophage Study

#### 2.13.1. Cell Culture and M1-like Macrophage Polarization

THP-1 cells were cultured in RPMI 1640 medium supplemented with 10% fetal bovine serum (S1480, FBS, Biowest, Nuaillé, France) and 1% penicillin–streptomycin (10,000 U/mL, Gibco, Grand Island, NY, USA) in an incubator at 37 °C, 5% CO_2_. For immuno-polarization experiments, THP-1 cells were differentiated by PMA (100 ng/mL, 79346, Sigma-Aldrich, St. Louis, MO, USA) for 6 h, followed by the addition of GM-CSF (25 ng/mL, 130-093-865, Miltenyi Biotec, Bergisch Gladbach, Germany), LPS (100 ng/mL, L2762, Sigma-Aldrich, St. Louis, MO, USA), and IFN-y (20 ng/mL, 285-IF, R&D systems, Minneapolis, MN, USA) for 18 h.

#### 2.13.2. Inhibition of GOT1 by AOAA

In our experiment, we utilized two different cell types:

1. M1-like macrophages: Following differentiation, M1-like macrophages were treated with AOAA (1 mM) for 24 h in an incubator at 37 °C with 5% CO_2_. The treated macrophages were subsequently employed in further experiments.
2. PBMCs: Isolated PBMCs from rats were cultured in a medium containing 10% FBS and 1% P/S in the presence of AOAA (5 mM), LOLA (100 μg/mL), and ASP (2 mM). The cells were cultured for 20–22 h in an incubator at 37 °C with 5% CO_2_. On the following day, these cultured cells were used in further experiments.

#### 2.13.3. Inhibition of Got1 by siRNA

Got1 siRNA transfection was performed in 1.0 × 10^6^ M1-like macrophages using Lipofectamine RNAiMAX (Invitrogen, 13778, Carlsbad, CA, USA) following the manufacturer’s instructions. After a 4 h incubation, 10% FBS was added, and further experiments were conducted following an additional 24 h incubation period. The primer used in this study was as follows: Got1 siRNA; 5′-AACAGGUGCACUUCGAAUUGGUU-3′.

#### 2.13.4. Inhibition of Electron Transfer Chain

In our experiment, we utilized two different cell types.

1. M1-like macrophages: The cells were treated with specific ETC inhibitors, including ETC inhibitor I (Rotenone, R8875, 120 nM), ETC inhibitor III (Antimycin A, A8674, 30 nM), and ETC inhibitor V (Oligomycin, O4876, 100 nM) for a duration of 24 h. All reagents used in the experiment were purchased from Sigma-Aldrich (St. Louis, MO, USA).
2. PBMCs: Isolated PBMCs were cultured in a medium containing 10% FBS and 1% P/S for overnight in an incubator at 37 °C with 5% CO_2_. On the following day, the cells were treated with ETC inhibitors (Rotenone, Antimycin A, and Oligomycin, each at 20 nM) and co-treated with LOLA (100 μg/mL) and ASP (2 mM), each in its presence or absence for 3 h. Subsequently, these cultured cells were used in further experiments.

### 2.14. Human PBMC Study (ASP Level and Patients’ Outcome)

This study was approved by the institutional review board of CHA University Bundang Medical Center (IRB No. 2020-06-042). Human blood samples were obtained from patients with sepsis defined as Sepsis-3. Written informed consent was obtained from all patients prior to sampling.

PBMCs were isolated from human peripheral blood (septic patients and healthy volunteers) using density gradient centrifugation. Fresh heparinized blood was placed into 50 mL conical centrifuge tube, to which an equal volume of room-temperature PBS was added, and this was mixed by inverting the tube several times. The diluted blood sample was carefully layered onto an equal volume of Ficoll-Paque PLUS (Cytiva, Uppsala, Sweden) and centrifuged at 400× *g* for 20 min at 20 °C without break. The PBMC layer was transferred to another tube and centrifuged at 300× *g* for 5 min at 20 °C to obtain PBMC pellets. To remove any remaining red blood cells (RBCs), the cell pellet was resuspended in 1 mL RBC lysis buffer for the human blood (J62990, Thermo Fisher, Waltham, MA, USA) for 5 min at room temperature, and then diluted with 9 mL of PBS. The mixture was centrifuged at 300× *g* for 5 min at 4 °C and the supernatant was removed. Finally, to wash the cell pellet, the pellet was resuspended in 5 mL of PBS and centrifuged at 300× *g* for 5 min at 4 °C two times.

Aspartate levels were determined using the Aspartate assay kit (ab102512, Abcam Waltham, MA, USA). Cell lysates were obtained by centrifuging at 14,000× *g* for 15 min at 4 °C, and the resulting supernatant was collected. Subsequently, lysates were incubated with the enzyme reaction mixture at room temperature, protected from light. The absorbance was measured at 570 nm, and the aspartate concentration was calculated using the following equation: Aspartate concentration (μM) = sample amount from the standard curve (nmole)/the volume of the sample added to the wells (μL).

### 2.15. Cytokine Measurements

The levels of the cytokine TNF-α (rat: ab236712, human: ab181421, Abcam, MA, USA) in the splenocytes, PBMCs and THP-1 cell were measured using ELISA kits according to the manufacturer’s instructions. The optical density at 450 nm was detected by a microplate reader (VersaMax with SoftMax Pro 7.1.1 software, Molecular Devices, San Jose, CA, USA).

### 2.16. Statistical Analysis

Normality was assessed using the Shapiro–Wilk test. Normally distributed data are presented as mean ± SD and were compared using independent t-tests or one-way ANOVA with appropriate post hoc testing. Non-normally distributed data are presented as median (IQR) and were compared using Mann–Whitney U or Kruskal–Wallis tests. Survival was analyzed with Kaplan–Meier curves and the log-rank test. Correlations were assessed using Spearman’s rank correlation. Two-sided *p* values < 0.05 were considered statistically significant. Analyses were performed using Sigma Plot software (ver 14.5, SYSTAT Software, San Jose, CA, USA).

## 3. Results

### 3.1. Sepsis Induces Endotoxin Tolerance and Impairs Phagocytosis

**Figure 1:**
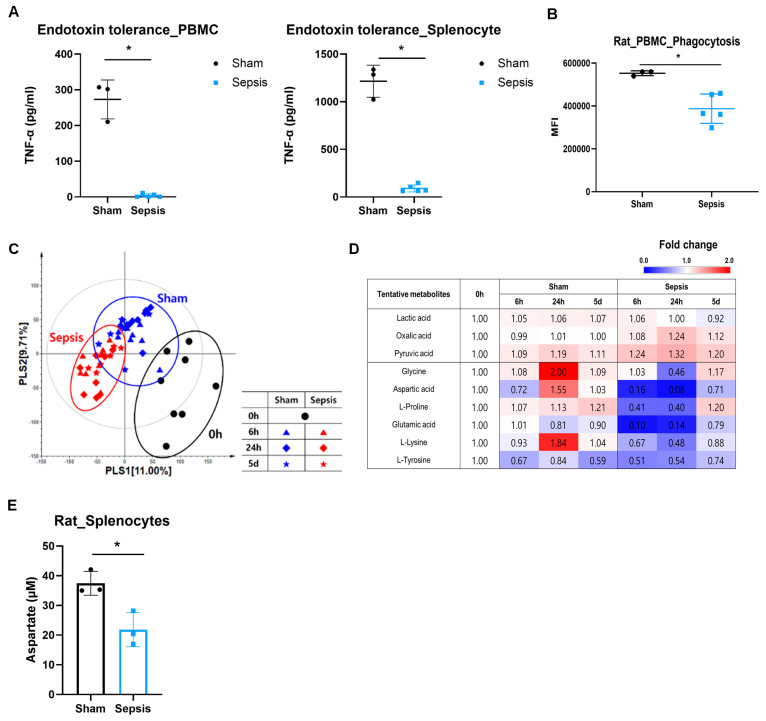
ASP deficiency is associated with the immune suppression in in vivo sepsis model. (**A**) Endotoxin tolerance in sepsis. PBMCs and splenocytes were isolated from sham and sepsis-induced rats, and supernatant was collected after LPS co-treatment for ELISA. Data represented as mean ± SD (sham n = 3, sepsis n = 5, Mann–Whitney U test, * *p* < 0.05). (**B**) Phagocytosis in sepsis. Isolated PBMCs were cultured with *E. coli*-FITC and assessed for phagocytosis activity by flow cytometry. (sham n = 3, sepsis n = 5, Mann–Whitney U test, * *p* < 0.05). (**C**,**D**) Metabolomic profiling of PBMCs in serial sepsis model. C Partial least-squares discriminant analysis score plot. (**D**) Heatmap analysis. ASP level was decreased in sepsis. (**E**) ASP level was measured by colorimetric detection method in splenocytes from sham and sepsis-induced rat. (n = 3 per group, two-sample *t*-test, * *p* < 0.05).

At 24 h after sepsis induction, both PBMCs and splenocytes exhibited reduced TNF-α production after LPS stimulation, consistent with endotoxin tolerance (Figure 1A). PBMC phagocytic activity was also significantly reduced in septic animals compared with sham controls (Figure 1B).

### 3.2. Metabolomics Identifies Intracellular Aspartate Depletion During Sepsis

GC-TOF-MS and UHPLC–LTQ–Orbitrap–MS/MS profiling of PBMCs demonstrated a clear separation between sham and septic groups across the time course in multivariate analyses (Figure 1C, Figures S1 and S2). These findings indicate broad remodeling of endogenous metabolites during sepsis.

### 3.3. Heatmap and Pathway Analyses Implicate Amino-Acid Biosynthesis Pathways

Using VIP-based feature selection (VIP > 0.7; *p* < 0.05), we generated a heatmap of discriminatory metabolites (Figure 1D). Several amino-acid pathways, including those involving glycine, lysine, glutamate, aspartate, and proline, were downregulated in septic PBMCs (Figure S3).

### 3.4. Intracellular Aspartate Is Reduced in Splenocytes After Sepsis

To confirm ASP levels in sham and sepsis group, we measured aspartate levels in rat splenocytes with ELISA method. We observed a 26% decrease in intracellular aspartate in sepsis compared to sham (Figure 1E).

### 3.5. ASP Supplementation Increased Survival and Mitigated Acute Kidney Injury (AKI) After Sepsis

**Figure 2:**
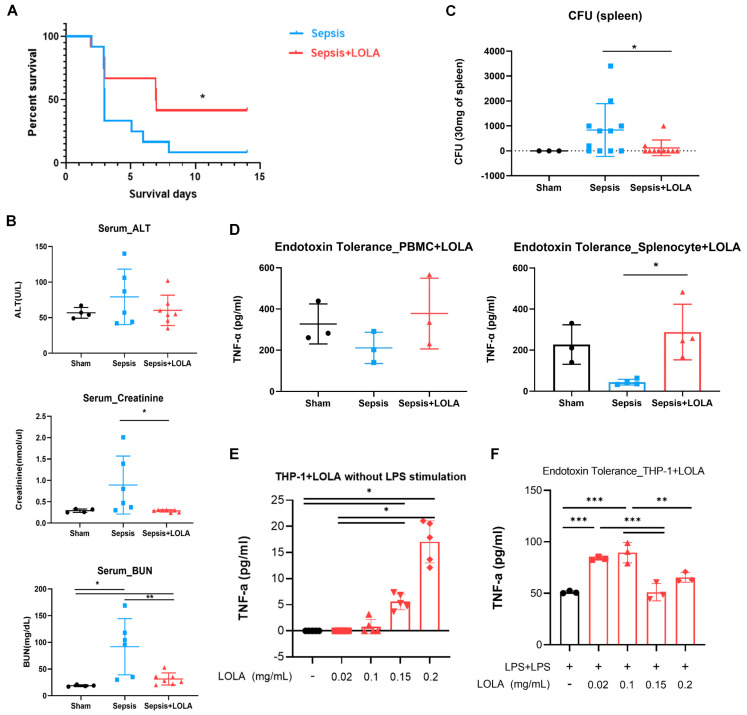
Effects of supplementation of ASP on sepsis model, in vivo and in vitro model. (**A**) Effects of LOLA supplementation in polymicrobial sepsis model, survival rate (sepsis n = 12, sepsis + LOLA n = 11, Kaplan–Meier log-rank test, * *p* < 0.05). LOLA; L-ornithine L-aspartate. (**B**) Organ dysfunction was analyzed in serum levels of B-1 ALT, B-2 Creatinine, B-3 BUN (sham n = 4, sepsis n = 6, sepsis + LOLA n = 7, Kruskal–Wallis test, * *p* < 0.05, Mann–Whitney U test, ** *p* < 0.05). (**C**) Bacterial load in spleen tissue (sham n = 3, sepsis n = 11, sepsis + LOLA n = 10, Kruskal–Wallis test, NS, Mann–Whitney U test, * *p* < 0.05), CFU; colony-forming unit. (**D**) Ex vivo endotoxin tolerance. PBMCs and splenocytes from sham, sepsis-induced, or LOLA-injected rats were stimulated by LPS before harvest of supernatant and measured using ELISA. (Sham n = 3, sepsis n = 3–4, sepsis + LOLA n = 3–4, one-way ANOVA, * *p* < 0.05). (**E**,**F**) Effects of LOLA on TNF-α production in in vitro THP-1 cells. THP-1 cells were cultured with different concentrations of LOLA (0.02–0.2 mg/mL). E In the absence of LPS (n = 5 per group, Kruskal–Wallis test, * *p* < 0.05). (**F**) In the presence of LPS (endotoxin tolerance; n = 3 per group, one-way ANOVA, ** *p* < 0.01, *** *p* < 0.001).

ASP supplementation with L-ornithine L-aspartate (LOLA) after sepsis increased survival rate in sepsis from 12.5% to 37.5% (Figure 2A), and decreased AKI shown in serum creatinine. However, there is no change in ALT, meaning there is no difference in liver injury (Figure 2B). There are no significant differences in glucose, albumin, and electrolyte with LOLA supplementation (Figure S4).

### 3.6. ASP Supplementation Enhances Bacterial Clearance

ASP supplementation significantly reduced the bacterial burden in the spleen compared with untreated septic animals (Figure 2C).

### 3.7. LOLA Enhances Cytokine Responsiveness in Ex Vivo and In Vitro Endotoxin Tolerance Models

In ex vivo assays, PBMCs and splenocytes from LOLA-treated animals produced higher TNF-α after LPS stimulation than cells from untreated septic animals (Figure 2D). In THP-1 endotoxin tolerance assays, LOLA dose-dependently increased TNF-α production both in the absence and presence of LPS (Figure 2E,F).

### 3.8. Transcriptomics Implicates Reduced GOT1 Expression in Impaired ASP Synthesis

For the interpretation of the decrease in intracellular ASP levels in sepsis, we conducted a transcriptome comparison analysis using RNA-seq (Table S1) between sham and sepsis.

**Figure 3:**
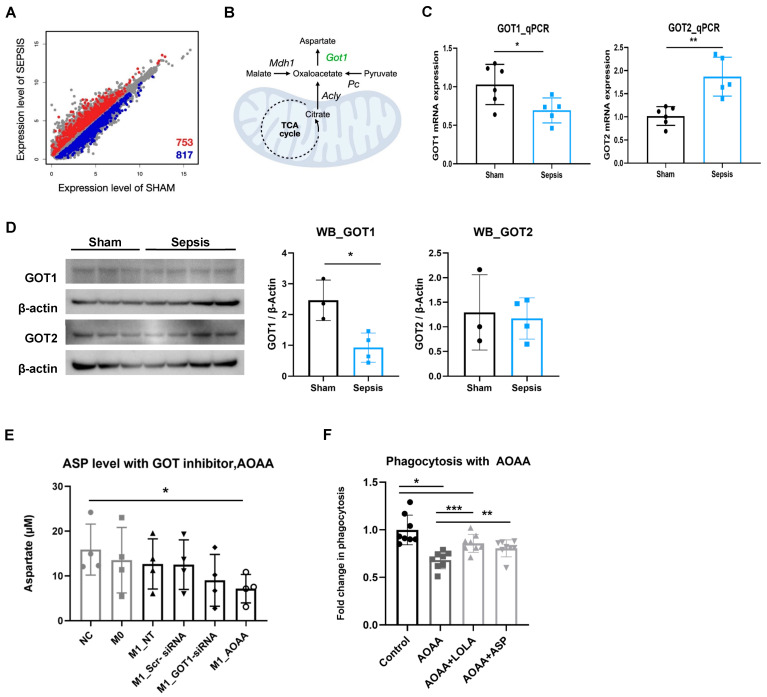
The role of ASP production in immune function in sepsis (**A**,**B**) The model for screening enzymes related to ASP production in sepsis. (**A**) The scatter plot of differentially expressed enzymes between sepsis and sham. (**B**) Aspartate biosynthetic pathway. Green color indicates an enzyme lowly expressed in the sepsis. (**C**) The mRNA expression of Got1/2 in splenocytes measured by RT-qPCR (Sham n = 6, Sepsis n = 5, Two sample *t*-test, * *p* < 0.05, ** *p* < 0.01). (**D**) Western blot measurements of GOT1/2 protein levels in splenocytes. β-Actin served as the loading control (sham n = 3, sepsis n = 4, two-sample *t*-test, * *p* < 0.05). (**E**) ASP level was measured by colorimetric detection method in 1 mM AOAA or 25 pmol Got1 siRNA-treated M1-like macrophages. (n = 4 per group, two-sample *t*-test, * *p* < 0.05). NC; normal control (THP-1), M0; M0-like macrophage, M1; M1-like macrophage, NT; non-treated, Scr-siRNA; scrambled siRNA. (**F**) PBMCs were cultured with AOAA, LOLA, or ASP and further incubated with *E. coli*-FITC. Phagocytosis of PBMCs was analyzed by flow cytometry, and data are shown as fold change in phagocytic cells. (n = 8 per group, one-way ANOVA, * *p* < 0.05, two-sample *t*-test, ** *p* < 0.05 *** *p* < 0.01).

Because the ASP level can be influenced by enzymes that utilize ASP as a substrate or produce ASP as a product, we focused on the genes specifically encoding enzymes classified within EC (enzyme commission) system. We identified 1570 differentially expressed enzymes (DEEs) at significance level 0.01 (Figure 3A) and further performed their Gene Ontology (GO) analysis.

Among the enriched KEGG pathways of the DEEs, the alanine, aspartate, and glutamate metabolism pathway (KEGG ID: hsa00250) was observed to be significant (FDR = 6.21 × 10^−5^). Within the Asp biosynthetic pathway, GOT1 expression was significantly reduced in sepsis, whereas several upstream enzymes (Mdh1, Acly, and PC) did not meet criteria for differential expression (Figure 3B).

### 3.9. GOT1 Suppression Reduces ASP Availability and Impairs Phagocytosis

qPCR confirmed decreased Got1 mRNA and increased Got2 mRNA in sepsis (Figure 3C). Western blotting showed reduced GOT1 protein levels in sepsis splenocytes, with no significant change in GOT2 (Figure 3D). Pharmacologic (AOAA) or siRNA-mediated GOT1 inhibition reduced intracellular ASP in M1-like macrophages (Figure 3E). In rat PBMCs, AOAA reduced phagocytosis, which was partially restored by LOLA or ASP supplementation (Figure 3F).

### 3.10. ETC Inhibition Reduces ASP and Decreases Phagocytosis

**Figure 4:**
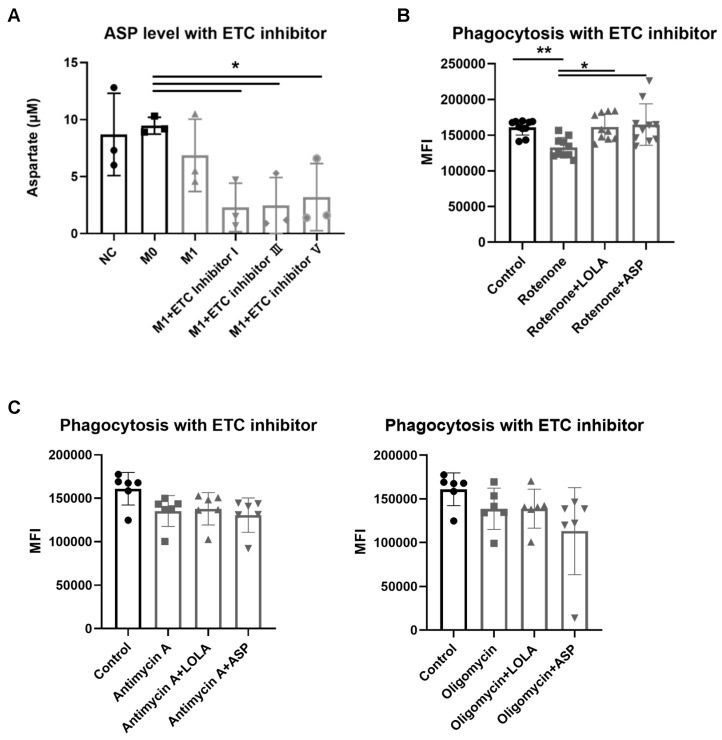
The role of ETC to supply ASP in immune function (**A**) M1-like macrophages were treated with 120 nM ETC inhibitor I, 30 nM ETC inhibitor III, or ETC inhibitor V. To assess ASP levels, cells were lysed, and the collected supernatant was used for aspartate assay. (n = 4 per group, two-sample *t*-test, * *p* < 0.05). NC; normal control (THP-1), M0; M0-like macrophage, M1; M1-like macrophage. (**B**,**C**) Effects of LOLA and ASP on phagocytosis. Isolated PBMCs from rat whole blood were cultured overnight and treated with (**B**) Rotenone, (**C**) Antimycin A, or Oligomycin each at 20 nM and LOLA or ASP. Phagocytosis activity was assessed using flow cytometry after further incubation with *E. coli*-FITC. ((**B**); n = 10 per group, C; n = 6 per group, one-way ANOVA, * *p* < 0.05, ** *p* < 0.01).

We also confirmed the effect of ETC inhibition in M1-like macrophages, resulting in a significant decrease in aspartate levels. The ETC inhibitors I (Rotenone, 120 nM), III (Antimycin A, 30 nM), and V (Oligomycin, 100 nM) decreased approximately 71%, 69%, and 59%, respectively, compared to the untreated group (Figure 4A). Then, we investigated the phagocytic capacity of LOLA and ASP under ETC-inhibited rat PBMCs. Treating rat PBMCs with ETC inhibitors (Rotenone, Antimycin A, Oligomycin, each at 20 nM) led to a decrease in phagocytosis of 17%, 14%, and 25%, respectively. The LOLA administration exhibited an approximately 22% increase in phagocytosis compared to the Rotenone group. Similarly, the ASP group showed an enhancement of 23% in phagocytosis compared to the Rotenone group (Figure 4B). However, the LOLA or ASP under Antimycin A or Oligomycin treatment did not significantly change the phagocytosis (Figure 4C). These results suggest that LOLA and ASP enhance phagocytic activity specifically in Rotenone-treated PBMCs.

### 3.11. Intracellular Aspartate Is Decreased in PBMCs from Patients with Sepsis

**Figure 5:**
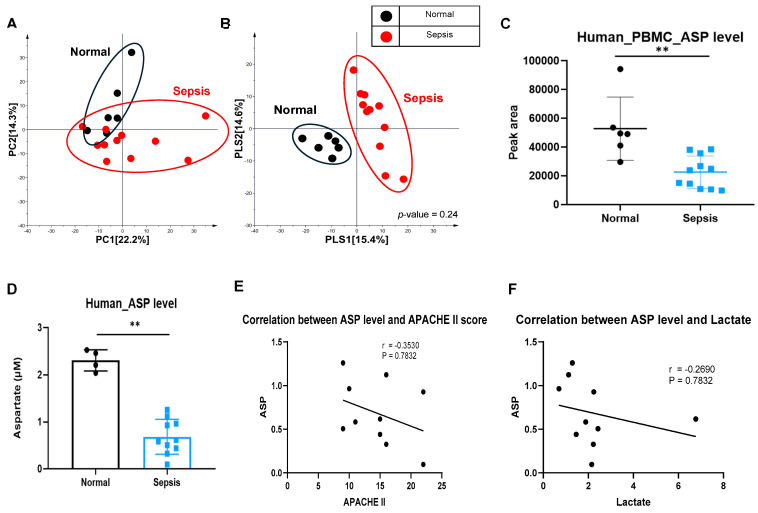
ASP level in human PBMCs was decreased in sepsis. (**A**,**B**) Metabolic differences between PBMCs of healthy volunteers and septic patients. (**A**) Principal component analysis (PCA) score plot. (**B**) Partial least-squares discriminant analysis (PLS-DA) score plot derived from the GC-TOF-MS dataset. (**C**) ASP levels measured by LC–Triple-Quadrupole-MS in PBMCs from healthy volunteers and septic patients. (Normal n = 6, sepsis n = 11, two-sample *t*-test, ** *p* < 0.01). (**D**) ASP levels measured by colorimetric detection method in PBMCs from healthy volunteers and sepsis patients. (Normal n = 4, sepsis n = 10, two-sample *t*-test, ** *p* < 0.01). (**E**,**F**) Correlation with ASP level and severity of sepsis, (**E**) APACHE II or (**F**) lactate level (n = 10 per group).

Metabolomic profiling of human PBMCs showed distinct clustering between healthy volunteers and patients with sepsis in PCA and PLS-DA analyses (Figure 5A,B). Targeted LC-MS/MS and colorimetric assays consistently demonstrated lower intracellular Asp in PBMCs from patients with sepsis compared with healthy controls (Figure 5C,D). Asp levels showed non-significant inverse trends with APACHE II score and lactate (Figure 5E,F).

## 4. Discussion

This study integrates metabolomics and transcriptomics with functional validation to identify intracellular Asp depletion as a feature of sepsis-associated immune suppression. Across rat and human immune cells, lower intracellular Asp was associated with reduced cytokine responsiveness and impaired phagocytosis. In vivo supplementation with LOLA improved survival, enhanced bacterial clearance, and attenuated kidney injury.

We assessed intracellular ASP levels. Although plasma or serum concentrations are routinely measured in clinical practice, intracellular measurements may more directly reflect the functional effects of metabolites on cellular activity. In PBMCs and splenocytes from septic subjects, intracellular ASP levels were reduced, whereas ASP levels in serum and whole-spleen samples did not differ significantly (data not shown).

While ASP has been studied extensively in cancer—particularly for its roles in cell proliferation and survival—its role in sepsis remains relatively underexplored [12]. Cytosolic ASP levels have been shown to influence cell viability, and ASP depletion is associated with tumor growth arrest [26,27]. Moreover, ASP supplementation can support mitochondrial function and improve survival during anticancer drug treatment [28]. ASP metabolism has also been linked to angiogenesis [29]. Collectively, these findings suggest that cytosolic ASP is critical for cellular function, especially in highly proliferative and metabolically active cells. In line with this concept, our study demonstrates that reduced ASP levels in animal models of sepsis, or chemically induced ASP deficiency, are closely associated with immunosuppression.

Mitochondrial function is essential for sustaining ASP availability in proliferating cells, thereby supporting protein, RNA, and DNA synthesis [30,31]. In M1 macrophages, ASP and arginine are upregulated [32], and inhibition of GOT1 (e.g., with AOAA or a GOT1 inhibitor) reduces nitric oxide and IL-6 production, suggesting dampened inflammatory responses. Consistent with these observations, we found that GOT1 inhibition-induced immunosuppression was partially reversed by ASP supplementation, further supporting a functional role for ASP in immune regulation.

The immune response in sepsis is highly heterogeneous, spanning from excessive inflammation to profound immunosuppression [9]. This variability complicates the use of immunomodulatory therapies, and as a result, sepsis management remains largely supportive. Effective immune-directed treatment in sepsis will require a precision medicine approach, supported by reliable biomarkers. However, there is currently no routine clinical method to directly assess immune-cell functional status in sepsis. Laboratory assays such as endotoxin tolerance testing, PD-1/PD-L1 expression, or HLA-DR measurement can be informative, but their availability varies across institutions, and turnaround times may be too slow for a condition with hyperdynamic clinical course [33]. In this context, intracellular ASP levels in PBMCs may serve as a practical surrogate marker of immunosuppression and could offer a feasible option for routine monitoring.

ASP supplementation improved survival in an animal model of sepsis. Using LOLA to increase ASP availability may offer a clinically practical strategy, as LOLA is an FDA-approved therapy for liver dysfunction with an established safety profile, potentially enabling sepsis trials more readily than newly developed ASP-containing agents or drugs with greater toxicity. In our in vitro experiments, LOLA and ASP produced comparable immunologic effects, further highlighting ASP as the critical mediator.

LOLA has been shown to modulate innate immune responses as well as the fecal microbiome and metabolome in patients with liver cirrhosis, and similar mechanisms may also contribute to its beneficial effects in sepsis models [34].

In clinical practice, LOLA could be considered either in patients selected by low PBMC ASP levels or during the delayed phase of sepsis, which is typically characterized by immunosuppression.

In recent years, immunometabolism has emerged as an important framework for understanding sepsis pathophysiology and offers opportunities to develop metabolic biomarkers and biomarker-driven precision medicine approaches. Our study further supports and extends these concepts [35].

This study has several limitations. Firstly, we did not specify the subtype of splenocytes or PBMCs involved in the effects of ASP. T and B lymphocytes, monocytes, dendritic cells, and macrophages are all localized in the spleen and PBMCs [36], making it challenging to pinpoint the specific immune cells responsible for the immunomodulatory effects of ASP. Recently, cell-type-specific metabolic regulation of immune responses has received increasing attention, and our findings warrant further investigation and extension in this direction [37]. Secondly, for the sake of convenience in subsequent clinical trials, we utilized LOLA in the in vivo sepsis model instead of ASP itself. However, in our in vitro study, both LOLA and ASP exhibited the same immunological effects. Thirdly, aside from GOT, we did not explore other pathways associated with ASP metabolism. Despite these limitations, this study is the first to investigate the immunological effects of ASP on sepsis. Lastly, although cecal slurry and cecal ligation-and-puncture (CLP) models are among the most clinically relevant experimental models of sepsis [38], persistent gaps between preclinical models and human sepsis still complicate translation of our findings to real-world clinical settings. Cecal slurry model had advantage over CLP, in terms of reproducibility and scalability.

## 5. Conclusions

Intracellular ASP deficiency is associated with immune suppression in sepsis. ASP repletion with LOLA improved immune function and survival in a polymicrobial rat sepsis model. This finding was consistent with reduced ASP levels in PBMCs from patients with sepsis. This suggests that ASP has the potential to serve as both a novel biomarker and a therapeutic target for immune suppression in sepsis.

## Data Availability

The datasets generated and analyzed during the current study are available from the corresponding authors upon reasonable request due to patent.

## Supplementary Materials

The following supporting information can be downloaded at https://www.mdpi.com/article/10.3390/cells15060513/s1. Figure S1: Principal component analysis (PCA) score plot derived from the GC-TOF-MS dataset of PBMCs. Figure S2: (A) PCA score plot; (B) PLS-DA score plot derived from the UHPLC-LTQ-Orbitrap-MS/MS dataset of PBMCs. Figure S3: Metabolic pathways and changes in discriminant metabolites in PBMCs; Figure S4: Effects of ASP supplementation in a polymicrobial sepsis model on blood chemistry and electrolytes (S4-A, Sham = 3, Sepsis = 3, Sepsis + LOLA = 5, Kruskal-Wallis test), (S4-B, Sham = 3, Sepsis = 3, Sepsis + LOLA = 5, One-way ANOVA, * *p* < 0.05); Table S1: RNA-seq analysis summary statistics.
