# Supplementary material for: Role of Aspartate in Immune Response and Mortality in a Polymicrobial Sepsis Model: Insights from Metabolomics and Transcriptomics
Source: Cells. 2026 Mar 13;15(6):513. doi: 10.3390/cells15060513 (PMC13025803; doi:10.3390/cells15060513)
Supplement: Supplementary file 1 [file cells-15-00513-s001.zip › cells-4036211-supplementary.pdf]

Title page

Title: Role of Aspartate in Immune Response and Mortality in Sepsis: Insights from Metabolomics and Transcriptomics

Authors: Min Ji Lee[1,#], Bo Mi Kim [1,#], Se Rin Choi [2,#], Seongmin Kim[3], Ye Jin Park[1], Yun-seok Kim[1], Kihwan Choi[1], Chang June Yune[1], Tae Nyoung Chung[1], Jinkun Bae[1], Nam Joo Yun[1], Jiwon Jeon[1], Han A Reum Lee[1], Jiewan Kim[1], Dong-Hyuk Kim[2], Ji Heon Noh[4], Chungoo Park[3], Sangchun Choi[5], Choong Hwan Lee[2,*], and Kyuseok Kim[1,*]

[1]Department of Emergency Medicine, CHA University School of Medicine, Gyeonggi-Do, Republic of Korea.

[2]Department of Bioscience and Biotechnology, Konkuk University, Seoul, Republic of Korea.

[3]School of Biological Science and Technology, Chonnam National University, Gwangju, Republic of Korea.

[4]Department of Biochemistry, Chungnam National University, Daejeon, Republic of Korea.

[5]Emergency Department, Soonchunhyang University College of Medicine, Gyeonggi-Do, Republic of Korea.

[#] Min Ji Lee, Bo Mi Kim, and Se Rin Choi contributed equally to this article.

[*]Corresponding Authors: Choong Hwan Lee (chlee123@konkuk.ac.kr) and Kyuseok Kim (dremkks@cha.ac.kr or dreinstein70@gmail.com)

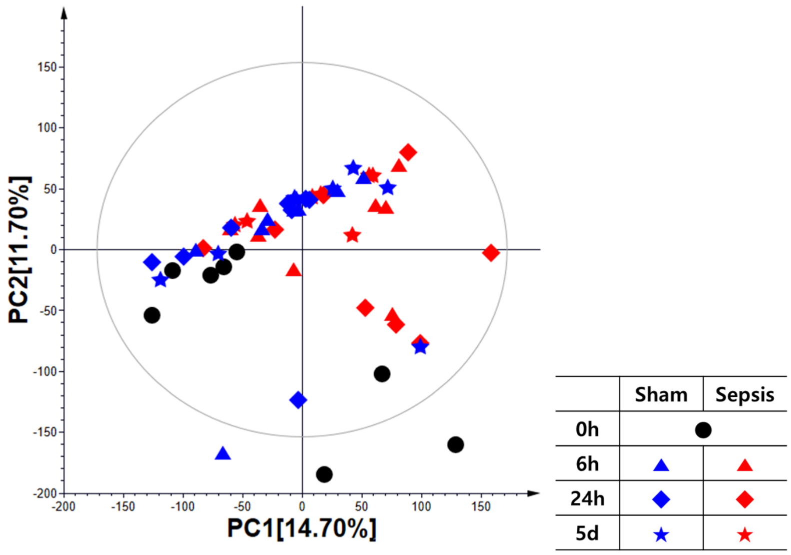

Figure S1 Principal component analysis (PCA) score plot derived from the GC-TOF-MS dataset of PBMCs.

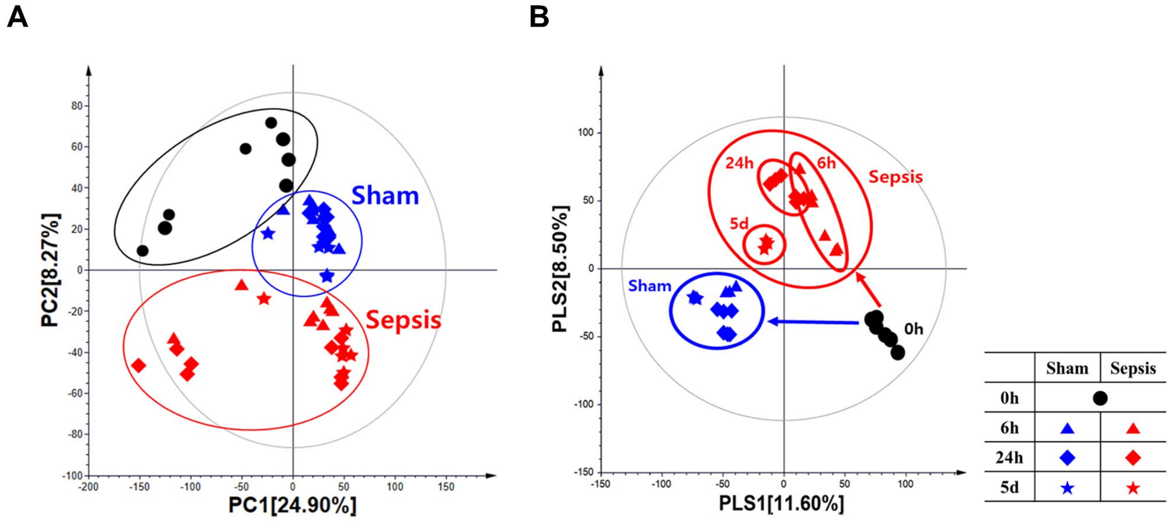

Figure S2 (A) PCA score plot; (B) PLS-DA score plot derived from the UHPLC-LTQ-Orbitrap-MS/MS dataset of PBMCs.

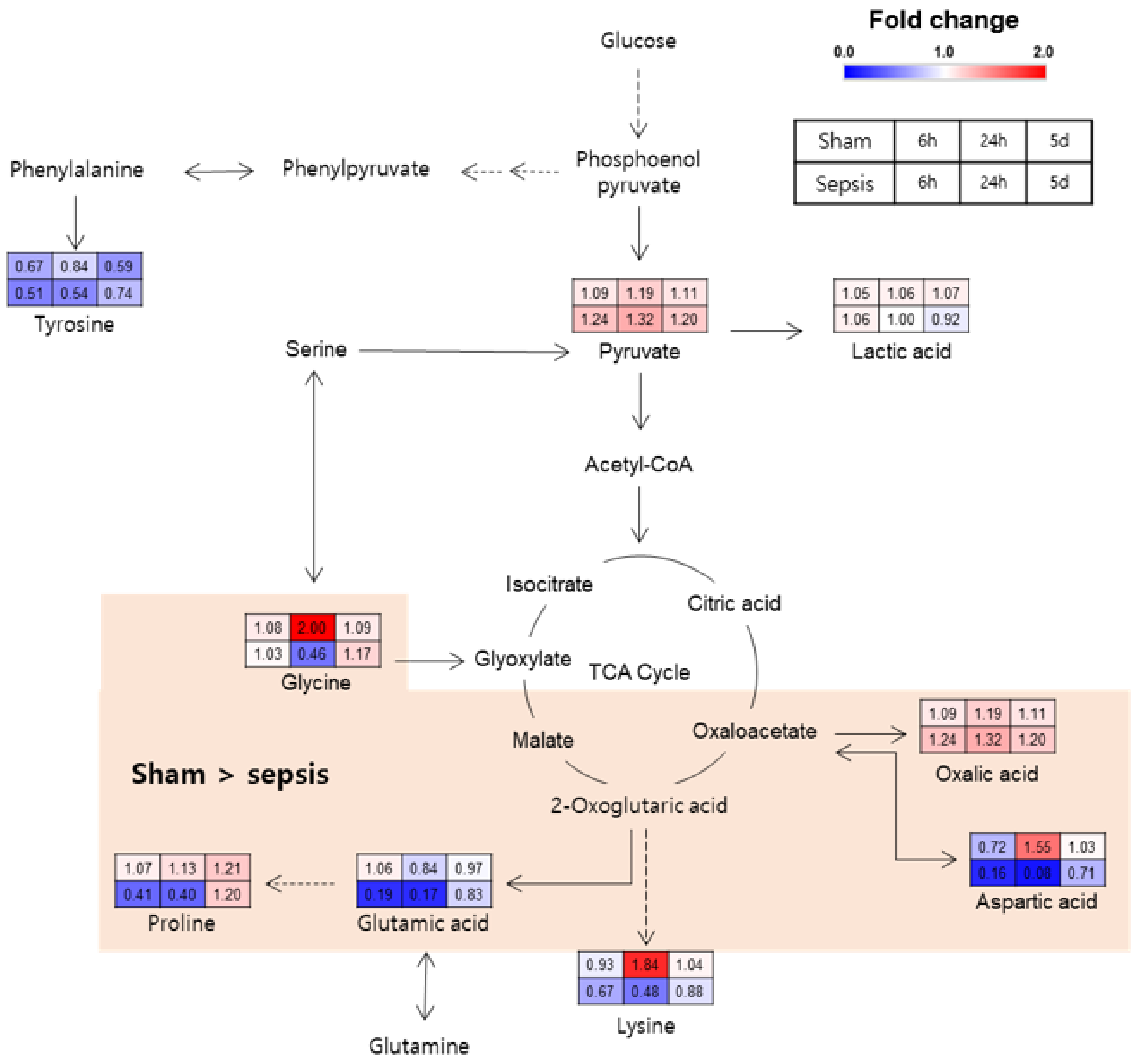

Figure S3 Metabolic pathways and changes in discriminant metabolites in PBMCs.

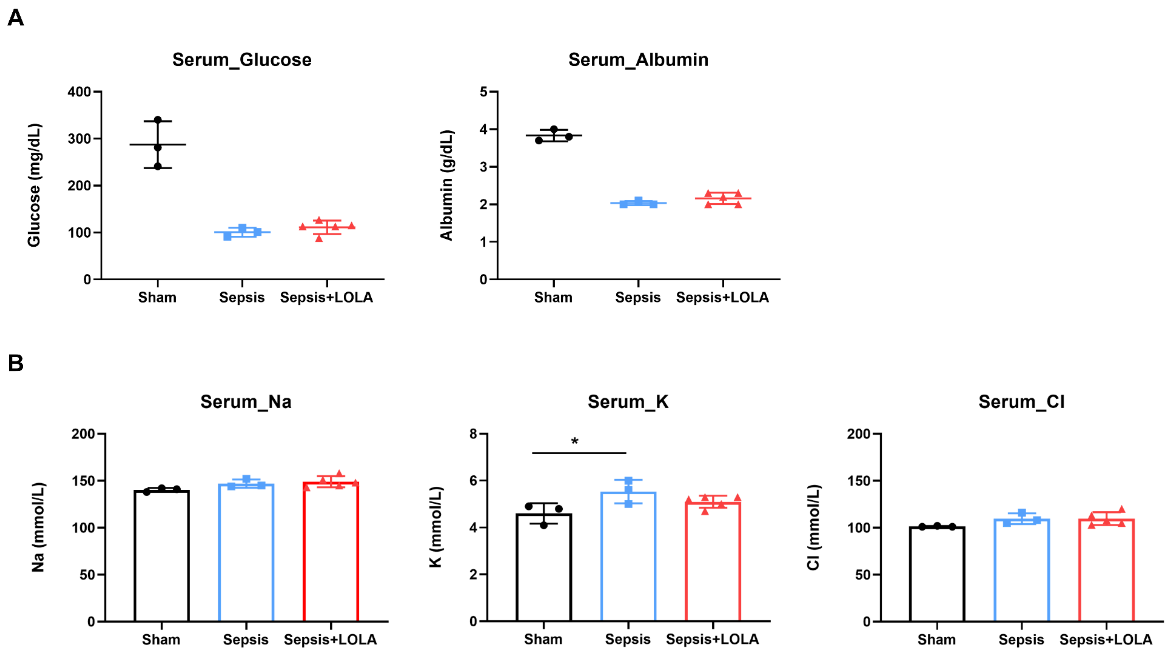

Figure S4. Effects of ASP supplementation in a polymicrobial sepsis model on blood chemistry and electrolytes (S4-A, Sham=3, Sepsis=3, Sepsis+LOLA=5, Kruskal-Wallis test), (S4-B, Sham=3, Sepsis=3, Sepsis+LOLA=5, One-way ANOVA, *$p < 0.05$).

Sample	Replicate	Number of Reads (Sum of Pairs)	Number of Reads after Trimming	Alignment Rate (%)
SHAM	1	62,897,298	61,501,892	97.92
	2	52,683,764	51,227,740	97.83
	3	42,072,978	40,716,902	97.64
	4	50,498,112	48,824,828	97.77
	5	58,110,284	56,235,544	97.49
	6	46,880,794	45,677,320	97.85
	7	62,426,236	60,865,762	97.89
	8	58,346,842	56,982,916	97.87
SEPSIS	1	51,112,394	49,633,888	97.83
	2	56,013,230	54,408,724	97.81
	3	39,931,406	38,933,498	97.34
	4	51,865,950	50,019,782	97.78
	5	49,415,906	47,574,124	97.76
	6	42,907,790	41,552,740	97.79
	7	54,758,540	52,893,366	97.92
	8	57,989,094	55,906,548	97.39

Table S1 RNA-seq analysis summary statistics.
